# Secondary Postpartum Hemorrhage Following Vaginal Birth After Cesarean (VBAC) Successfully Managed With the JADA® System

**DOI:** 10.7759/cureus.103689

**Published:** 2026-02-15

**Authors:** Preston A Phommathep, Kaitlyn Santos, Mackenzie Prosser, Shruthi Suresh, Hope Woodroffe

**Affiliations:** 1 Osteopathic Medicine, Lake Erie College of Osteopathic Medicine, Erie, USA; 2 Obstetrics and Gynecology, Sisters of Charity Hospital, Buffalo, USA

**Keywords:** intrauterine balloon tamponade, jada device, obstretic haemorrhage, postpartum hemorrhage, retained intrauterine clot, uterine atony, vaginal birth after cesarean section (vbac)

## Abstract

Postpartum hemorrhage (PPH) remains a significant cause of maternal morbidity, most commonly resulting from uterine atony. Management typically follows a stepwise approach using uterotonics and mechanical interventions when bleeding persists. We report the case of a 25-year-old G8P4043 (eight pregnancies, four carried to term, 0 preterm deliveries, four abortions, and three living children) woman at 38 weeks and three days’ gestation who underwent a successful vaginal birth after cesarean complicated by a 45-second shoulder dystocia. Initial quantitative blood loss was 300 mL with a firm uterus. Approximately two hours postpartum, the patient developed acute uterine atony with severe pain and heavy vaginal bleeding, resulting in a cumulative blood loss of 2,055 mL. Emergency bedside ultrasound demonstrated a markedly thickened endometrial stripe with a large intrauterine clot burden and minimal vascularity. Despite uterine massage, high-dose oxytocin, misoprostol, and repeated bimanual clot evacuation, the uterus remained boggy. Second-line uterotonics were deferred due to severe hypertension and a history of significant asthma. A vacuum-induced uterine hemorrhage control device, JADA® System (Organon & Co., Jersey City, NJ, USA), was placed following manual evacuation of intrauterine clots, resulting in rapid improvement in uterine tone and bleeding. The device was removed approximately five hours later without complication. Hemoglobin declined from 11.6 g/dL to a nadir of 8.3 g/dL, and no blood transfusion or surgical intervention was required. This case highlights the importance of early recognition, bedside ultrasound assessment, and timely escalation to mechanical therapy in the management of severe primary PPH, particularly in high-risk patients with contraindications to standard second-line uterotonic agents.

## Introduction

Postpartum hemorrhage (PPH), defined as blood loss of ≥1,000 mL within 24 hours of vaginal or cesarean delivery, remains a leading cause of preventable maternal morbidity and mortality. Uterine atony is the most common etiology [1,2]. PPH occurs in 4%-11% of pregnancies and is a leading cause of maternal mortality worldwide; however, in settings where prophylactic uterotonics are routinely administered, rates decrease to approximately 1.2% [3,4]. Established risk factors include prior cesarean delivery with placenta previa or accreta, placenta previa without prior cesarean delivery, and antepartum hemorrhage such as placental abruption [5]. Prevention follows a stepwise approach beginning with active management of the third stage of labor, including uterine massage and controlled umbilical cord traction. Although uterine massage remains a mainstay of management, it is not reliably preventative and is therefore used as an adjunct rather than a definitive measure [6,7].

Standard pharmacologic treatment includes uterotonics such as oxytocin, methylergonovine, and carboprost; however, maternal comorbidities such as hypertension and asthma may limit their use. When uterotonics are insufficient, escalation to mechanical or procedural interventions is required. Intrauterine balloon tamponade (e.g., Bakri balloon) and uterine compression sutures are commonly employed but may be less effective in the presence of significant intrauterine clot burden or may require operative intervention. Vacuum-induced hemorrhage control devices, such as the JADA® System (Organon & Co., Jersey City, NJ, USA), promote rapid uterine collapse to achieve bleeding control and have emerged as alternative therapies when pharmacologic options are limited or ineffective [6,7]. However, their precise role within the therapeutic algorithm, particularly in cases complicated by a large retained clot burden, remains incompletely defined. We present a case of severe PPH due to uterine atony with substantial retained clot burden successfully managed with the JADA® device after failure of second-line uterotonics.

## Case presentation

Patient information

A 25-year-old G8P4043 (eight pregnancies, four carried to term, 0 preterm deliveries, four abortions, and three living children) female patient presented in labor at 38 weeks and three days’ gestation. Past obstetric history included one prior cesarean delivery followed by two successful vaginal births after cesarean (VBAC). No last menstrual period (LMP) was recorded for the index pregnancy. Her medical history was notable for multiple risk factors for PPH, such as multiparity, history of prior cesarean section, chronic hypertension (managed with nifedipine 30 mg), class III obesity (on aspirin 81 mg), active tobacco use, and shoulder dystocia at delivery. These supported heightened preparedness, such as intravenous (IV) access, uterotonics on hand, and bedside ultrasound availability.

Other pertinent information involved with the patient’s history was that she had asthma requiring twice daily Advair and rescue albuterol, depression, anxiety, and post-traumatic stress disorder (PTSD) related to the prior death of a child. She also had a history of alcohol use disorder, including use during pregnancy, marijuana use during pregnancy, and prior suicidal/homicidal ideations with current Child Protective Services (CPS) involvement. Family history was significant for maternal breast cancer, hydrocephalus, and anemia, and the maternal grandmother had diabetes and pancreatic cancer. She reported an allergy to grapes. Medications included albuterol, acetylsalicylic acid (ASA) 81 mg, prenatal vitamins, escitalopram 20 mg, magnesium oxide 400 mg, nifedipine 30 mg, ondansetron 4 mg PRN, and rizatriptan.

Obstetric course and delivery

On September 22, 2025, at 38w3d, the patient noted thick brown/clear vaginal fluid earlier in the day, later described as clear and bloody, consistent with premature rupture of membranes at 11:00. On patient arrival at 18:00, her blood pressure (BP) was elevated at 156/77 mmHg. The patient noted she had not taken her scheduled Procardia 60 mg dose. Fundal height was deferred due to body habitus. Sterile speculum examination revealed bloody mucus, ferning, and nitrazine positive without pooling. Membranes were ruptured. Sterile vaginal examination demonstrated cervical dilation of 4 cm, 70% effacement, and a station of −3. The patient was augmented with Pitocin and proceeded with a trial of labor after cesarean (TOLAC). The patient pushed for 52 minutes with rupture of membrane (ROM)-to-delivery time of 10 hours and 22 minutes. She achieved spontaneous vaginal delivery (VBAC) at 21:22 without analgesia.

A 45 s right anterior shoulder dystocia complicated the delivery. After delivery of the fetal head, the right anterior shoulder failed to deliver, and McRobert’s maneuver, as well as suprapubic pressure and corkscrew maneuver, was performed to relieve the dystocia. A liveborn neonate was delivered at 21:22 with a birth weight of 3.445 kg (7 lb 9.5 oz). APGAR scores were 8 at one minute and 9 at five minutes. No immediate neonatal complications were documented.

The placenta was delivered at 21:26 on September 22, 2025, with active management during the third stage of labor, including uterine massage and controlled cord traction to decrease the chances of PPH from occurring. Initial quantitative blood loss (QBL) at delivery was documented as 300 mL. Maternal blood type was O positive. Perineal examination revealed a midline and left periurethral laceration, which was repaired with 4-0 Vicryl (Ethicon, Inc., Somerville, NJ, USA). The perineum, vagina, and cervix were otherwise intact. Fundal massage was performed, and the uterus was initially firm. Post-delivery medications included ketorolac 15 mg IV for analgesia, high-dose IV oxytocin per postpartum protocol, and misoprostol 800 mcg per rectum.

Postpartum deterioration

At approximately 23:16 (≈2 hours postpartum), the patient developed acute severe abdominal pain and marked distress, rolling side-to-side in bed. BP ranged from 166/86 to 183/98 mmHg despite confirmation with an appropriately sized cuff. On examination, the uterine fundus was 1 cm above the umbilicus and markedly boggy despite ongoing massage. A pad placed 10 minutes earlier was fully saturated. QBL increased rapidly from 300 mL at delivery to a cumulative 2,055 mL. Initial hemoglobin (Hb) and hematocrit (Hct) were 11.6 g/dL and 35.2%, respectively.

Emergent bedside ultrasound demonstrated an endometrial stripe measuring 4.7-4.9 cm with the uterine cavity filled with echogenic clot and minimal Doppler vascularity. Images were not retained due to the urgency of intervention. A second peripheral IV access attempt was unsuccessful. Although the patient remained hypertensive rather than hypotensive at this stage, the significant and rapidly escalating blood loss raised concern for impending hemodynamic compromise. Given preserved mentation, stable heart rate (HR), and absence of clinical signs of hypoperfusion at that time, arterial blood gas analysis was not obtained, as management proceeded emergently based on clinical findings.

Interventions

The patient was counseled and consented to intrauterine intervention. She was given 1 mg IV hydromorphone (Dilaudid) for analgesia. Sequential bimanual uterine exploration and massage were performed three times. Each pass expressed a large volume of clot. Despite aggressive manual clot evacuation and uterine massage, the uterus remained boggy and atonic. Two commonly used second-line uterotonics were deferred, which were methylergonovine and carboprost. Methylergonovine (Methergine) was withheld due to severe-range/elevated BPs up to 183/98 mmHg. Carboprost tromethamine (Hemabate) was withheld due to her significant asthma history (daily inhalers and active cigarette smoking) [6,8]. Given persistent atony with ongoing hemorrhage despite the use of uterotonics such as oxytocin/misoprostol, the decision was made to proceed with placement of a JADA® uterine vacuum device.

All appreciable intrauterine clots were first manually evacuated. The JADA® device was then inserted transcervically at 23:16. The cervical balloon was inflated with 120 mL of sterile water to create a seal, and the device was connected to continuous wall suction at 80 mmHg. A repeat bedside ultrasound confirmed the device in an appropriate intrauterine position, with a now thin endometrial stripe and no significant residual clot burden. Ultrasound imaging was not saved due to the need for quick interventions. Prophylactic cefoxitin 2 g IV was administered due to multiple bimanual intrauterine examinations and instrumentation. The patient’s subsequent Hb/Hct is 10.0 g/dL/30.3% at 23:50. The patient had 2 units of pure red blood cell (RBC) on hold in case they needed it, but the order was not transfused by the end of the case.

Outcome and follow-up

Following JADA® placement, vaginal bleeding rapidly improved. The device was left in place on suction. At 04:26 on September 23, 2025, the JADA® device was removed without difficulty. After removal, there was no further significant vaginal bleeding reported. At that time, the patient’s vitals were BP 148/72 mmHg, HR 111 bpm, Hb 8.3 g/dL, and Hct 25.0%. The lab values throughout the visit are reflected in Table 1, where the key phases were noted: baseline on admission, clinical deterioration (prior to JADA® placement), and post-intervention after JADA® removal. During clinical deterioration, elevations in BP and HR occurred in the setting of uterine atony and ongoing hemorrhage. Following placement of the JADA® vacuum-induced uterine tamponade device, vital signs stabilized, vaginal bleeding rapidly improved, and uterine tone normalized despite persistent anemia. These findings highlight the temporal relationship between intervention and clinical stabilization and underscore the utility of serial physiologic monitoring in guiding PPH management.

**Table 1:** Maternal Laboratory Results During the Postpartum Course mmHg: millimeters of mercury; bpm: beats per minute; g/dL: grams per deciliter; ABO: ABO blood group system; JADA®: vacuum-induced uterine tamponade device

Laboratory test	Baseline (admission)	23:26 (prior to JADA® placement)	04:26 (post-JADA® removal)	Reference range
Blood pressure (mmHg)	156/77	166/86-183/98	148/72	<120/80
Heart rate (bpm)	96	118	111	60–100
Respiratory rate (breaths/min)	18	24	18	12–20
Oxygen saturation (room air) (%)	98	97	99	≥95
Temperature (°C)	36.8	36.6	36.9	36.1–37.8
Hemoglobin (g/dL)	11.6	10.0	8.3	12.0–16.0
Hematocrit (%)	35.2	30.3	25.0	36.0–46.0
ABO blood group	O positive	O positive	O positive	Not applicable

On exam, the uterine fundus was firm at the level of the umbilicus. A perineal pad worn over the preceding 30 minutes showed only minimal lochia. The patient was able to void spontaneously. No blood transfusion administration or coagulation panel results were provided in the available documentation. The neonate remained stable throughout the maternal hemorrhage event. The patient was able to be discharged after one more night in the postpartum unit with close monitoring.

## Discussion

This case describes severe primary PPH shortly after delivery in a high-risk obstetric patient with multiple comorbidities and limited pharmacologic options. The patient initially had an uncomplicated immediate postpartum period with a firm uterus and QBL of 300 mL following VBAC, complicated by a brief shoulder dystocia. Within hours, however, she developed heavy vaginal bleeding, uterine atony, and a significant intrauterine clot burden with escalation of total QBL to 2,055 mL. Ultrasound was critical in demonstrating a distended uterine cavity with clot and guiding both clot evacuation and confirmation of device placement [9]. Due to the emergent nature of the case and institutional imaging workflow limitations, ultrasound images were not available for inclusion. The clinical course followed a clear time-dependent progression from initial stability (QBL 300 mL, firm uterus) to acute deterioration approximately two hours postpartum (QBL 2,055 mL, boggy uterus, and rising HR), prompting escalation to mechanical intervention.

Standard stepwise management of PPH due to atony generally includes uterine massage, high-dose oxytocin, additional uterotonics such as methylergonovine (contraindicated in uncontrolled hypertension) and carboprost (contraindicated in severe asthma), tranexamic acid, mechanical tamponade, fluid and blood product resuscitation, and surgical escalation if needed [10]. In this case, two key second-line uterotonics were relatively contraindicated. Methylergonovine was avoided due to severe-range postpartum hypertension, with BPs reaching 183/98 mmHg [6]. Carboprost was contraindicated given the patient’s significant asthma requiring daily inhaler therapy and active smoking history [6,8].

Two units of packed RBCs were cross-matched and made immediately available should transfusion become necessary. Despite a cumulative blood loss of 2,055 mL and a decline in Hb from 11.6 to 8.3 g/dL, transfusion was deferred because the patient demonstrated preserved end-organ perfusion. Although severe-range hypertension (up to 183/98 mmHg) may coexist with hypovolemia and potentially obscure compensated shock, serial assessments showed stable mentation, preserved oxygen saturation, improving respiratory rate after hemorrhage control, and no evidence of oliguria. HR increased from 96 to 118 bpm during peak hemorrhage and subsequently improved following intervention, suggesting physiologic compensation rather than decompensation. There were no clinical signs of hypoperfusion such as altered mental status, cool extremities, delayed capillary refill, or progressive tachycardia.

Given the absence of clinical indicators of tissue hypoxia or metabolic compromise, arterial blood gas analysis and lactate measurement were not obtained. Management decisions were therefore guided by dynamic vital sign trends and clinical examination rather than BP values alone. Rapid bleeding control following JADA® placement likely prevented progression to overt shock. For these reasons, transfusion was not indicated despite the nadir Hb of 8.3 g/dL.

Because of these limitations, conservative mechanical management with the JADA® uterine vacuum device was pursued. The JADA® functions by creating continuous low-level suction within the postpartum uterus while simultaneously maintaining cervical seal [11]. This promotes uterine collapse and evacuation of retained clot, both of which improve uterine tone and reduce bleeding [12]. In this patient, placement of the device after manual clot evacuation stabilized bleeding without escalation to operative management or transfusion [13,14]. Of note, prophylactic broad-spectrum antibiotic coverage (cefoxitin 2 g IV) was given in the setting of repeated bimanual intrauterine exploration and device placement. This reflects common practice in some institutions to reduce endometritis risk when multiple intrauterine manipulations are performed.

Intrauterine balloon tamponade, such as the Bakri balloon, has demonstrated a success rate of 87.1% in women with PPH due to uterine atony in a cohort of 243 patients, representing the highest success rate among the evaluated secondary causes of PPH. Higher success rates with the Bakri balloon were shown in cases where the PPH blood loss was <1,000 mL, while those above 1,000 mL had significantly lower rates [15,16]. Although uterine compression sutures demonstrate success rates exceeding 90%, their use requires operative intervention and escalation to the operating room [6]. The JADA® had a success rate of about 91% in women with PPH from vaginal birth, and the median time for placement from delivery of placenta was 31 minutes at bedside for vaginal birth [17]. Reported success rates are comparable to those described for balloon tamponade, with JADA® demonstrating effectiveness in cases with hemorrhage volumes up to 3,000 mL prior to placement [15,17]. The efficiency of placement, with confirmation through bedside ultrasound, and the lack of need to go to the operating room make the JADA® a promising minimally invasive alternative within the stepwise management of PPH.

The social and mental health context is also important in this case. The patient’s psychiatric history (PTSD, depression/anxiety, and prior self-harm/harm ideation), substance use in pregnancy (alcohol, tobacco, and marijuana), chronic hypertension, asthma, and class III obesity all increase her overall peripartum risk profile and her risk for complications such as PPH. Her ongoing CPS involvement and trauma history, including the loss of a six-year-old child, underscore the need for multidisciplinary postpartum follow-up: obstetrics, anesthesia, psychiatry, social work, and pediatrics [18].

## Conclusions

This case demonstrates successful management of PPH due to uterine atony using the JADA® vacuum-induced hemorrhage-control device as a second-line mechanical intervention when contraindications to methylergonovine (severe-range hypertension) and concerns regarding prostaglandin use (significant asthma) limited pharmacologic options. The device achieved rapid hemorrhage control through vacuum-induced uterine collapse, avoiding surgical escalation and blood transfusion in a high-risk VBAC patient. While intrauterine balloon tamponade remains an established alternative, reported success rates decline with increasing hemorrhage volume, whereas JADA® has demonstrated effectiveness in cases with blood loss up to 3,000 mL prior to placement. Favorable outcomes in this setting appear to depend on early recognition, appropriate patient selection, ultrasound guidance, and structured escalation of care. Overall, this case contributes to the growing body of evidence supporting vacuum-induced uterine collapse as a practical adjunct within the stepwise management of severe atony-related PPH, particularly in medically complex patients with limited pharmacologic options.
